# The Undersea and Hyperbaric Medical Society. A report on the annual scientific meeting 2012, Phoenix, AZ, USA June 21–23

**DOI:** 10.1186/2046-7648-1-14

**Published:** 2012-12-01

**Authors:** Michael H Bennett

**Affiliations:** 1 Department of Anaesthesia, Diving and Hyperbaric Medicine, Prince of Wales Hospital and University of New South Wales, Barker St, Randwick, New South Wales, 2031, Australia

**Keywords:** Diving, Hyperbaric oxygenation, Carbon monoxide poisoning, Traumatic brain injury

## Abstract

This is a report on the content of the 45th annual scientific meeting of the Undersea and Hyperbaric Medical Society (UHMS) in Phoenix AZ, USA last June 21-23, 2012. The UHMS is the major representative body for both physicians and scientists in diving and hyperbaric physiology and medicine.

## Scope of the meeting

The UHMS annual scientific meeting remains the premier forum for the presentation of both basic science and clinical work in the related fields of diving and hyperbaric medicine. The diving medicine and physiology stream has strong representation from the navies of the world, most notably the US Navy, while the physiology and medicine of hyperbaric oxygen (O_2_) exposure draws contributors from a wide range of academic institutions around the globe.

## Diving, decompression illness and dive accident management

The mechanisms by which bubbles produce the clinical syndrome of decompression illness (DCI) continue to receive attention. Building on previously published work from the lab of Steven Thom (Institute for Environmental Medicine, PA, USA), a number of groups presented a work further defining the role of endothelial and neutrophil activation, and the interaction of a range of immune mediators. The protective effect of hyperbaric oxygen preconditioning prior to decompression stress via heat shock proteins, as demonstrated in a rat model by Wang Xu (Faculty of Naval Medicine, Shanghai, China), further confirms the ability of hyperbaric oxygen exposure to induce anti-oxidant and anti-apoptotic effects. Thom demonstrated the production of pro-inflammatory micro particles (MPs) in the plasma of human volunteers, having previously demonstrated that the injection of such particles into naive mice reproduced the vascular injury associated with DCI. Interestingly, Thom was unable to show a positive correlation between the number of MPs and bubble numbers in the human volunteers, although there was such a correlation with β-2 integrin expression (an indication of platelet-neutrophil interactions), previously used as an index of decompression stress.

Fang and Bao (Institute of Naval Medical Research, Shanghai, China) similarly demonstrated elevated levels of circulating inflammatory cytokines after a provocative decompression, including IFN-γ, TNF-α and IL-6. These changes were associated with the activation of mitogen-activated protein kinase pathways – further evidence of the importance of the bubble-endothelium interactions that result in clinical symptoms of DCI.

The recent interest in the mechanism of swimming-induced pulmonary oedema has been generated by a number of reports of deaths in the diving population where pulmonary oedema on surfacing seemed the likely cause. This phenomenon received considerable attention at the meeting. Richard Moon (Duke University Medical Center, Durham, NC, USA) investigated 20 volunteers using transthoracic echocardiography during head-out immersion exercise in cold water. He confirmed a progressive increase in pulmonary artery pressure during the exercise period and concluded that this was most likely due to the decreased left ventricular compliance. We are getting closer to understanding how very fit young naval diving recruits can develop pulmonary oedema while maximally exercising in the water.

## Hyperbaric oxygen therapy

Thom presented the latest in his impressive body of work investigating the mechanism of action of hyperbaric oxygen therapy (HBOT). While it is now clear that HBOT inhibits neutrophil β-2 integrin function via a nitric oxide-mediated pathway of actin *S*-nitrosylation (SNO-actin), it remains something of a paradox that HBOT does not appear to cause immunocompromise more generally - in fact, quite the reverse. At this meeting, Thom presented work using mouse neutrophils to demonstrate that SNO-actin impedes its own eradication, but cell activation in immune surveillance follows a pathway that slows actin turnover, thus promoting normal β-2 integrin function. Each year we get a little closer to explaining the complex actions of this most reactive of elements - oxygen.

At a clinical level, HBOT is advocated for the treatment of acute carbon monoxide poisoning, but the evidence is disputed by many outside the field. Lin Weaver (Hyperbaric Medicine, University of Utah School of Medicine, UT, USA) is the author of the most methodologically sound randomised clinical trial in this area and a vigorous proponent of HBOT for these cases. He and his team presented a series of papers drilling further down into the nature and consequences of this poisoning. Eighteen children studied prospectively displayed a high incidence of complaints in memory, attention and concentration, headaches, sleep disturbance and a number of related disturbances in other areas, despite scoring relatively normally on standard neuropsychological testing. It seems that the current tests may have rather poor sensitivity in this age group. This finding contrasts with a high incidence of abnormal testing for vestibular and balance function in the absence of subjective complaints.

Finally, the question of the efficacy of HBOT in chronic, mild traumatic brain injury seems likely to be settled over the next couple of years. This problem is highly relevant to the US military given the high numbers of service members affected by ‘post-concussive syndrome’. Weaver outlined the situation and gave details of the three separate clinical trials underway, all funded by the military and all randomised, sham controlled and blinded to allocation. The first results should be published in about a year, and are highly anticipated.

## Abbreviations

DCI: Decompression illness; IFN-γ: Interferon- γ; IL-6: Interleukin-6; TNF-α: Tumour necrosis factor-α.

## Competing interests

The author declares no financial or non-financial competing interests.

